# From a Common Symptom to a Rare Diagnosis: A Case Report of Metastatic Solitary Fibrous Tumors

**DOI:** 10.7759/cureus.70944

**Published:** 2024-10-06

**Authors:** Mariana F Santos, Joana F Novo, Mafalda M Silva, Sara F Cândido

**Affiliations:** 1 Family Medicine, Unidade de Saúde Familiar (USF) Gago Coutinho, Unidade Local de Saúde (ULS) Estuário do Tejo, Alverca do Ribatejo, PRT

**Keywords:** metastasis, neoplasms, rare tumor, soft tissue tumor, solitary fibrous tumor

## Abstract

Low back pain is one of the most common reasons for medical appointments in primary care. Although mostly benign and self-limited, it requires careful evaluation in order to identify serious underlying conditions.

Solitary fibrous tumors (SFTs) are rare fibroblastic mesenchymal neoplasms with low metastatic potential that can be present anywhere in the body, with nonspecific associated symptoms.

We exhibit the case of a young patient who was diagnosed with metastatic SFT after perceiving a painless mass in the dorsal region associated with low back pain refractory to analgesics, describing its features, and performing a literature review.

## Introduction

Low back pain is a common reason for medical appointments in primary care, with an estimated 60-80% of people experiencing it at some point throughout their lives [1]. Generally, low back pain is benign and self-limited, making it crucial for physicians to be aware of red flags that indicate more serious conditions, thus decreasing morbidity and associated mortality.

Solitary fibrous tumors (SFTs) comprehend a spectrum of rare fibroblastic mesenchymal neoplasms that arise in serosal membranes, the meninges, and deep soft tissues with low metastatic potential [2-4]. In the contemporary series, about 30% of tumors arise in the thoracic cavity (pleura, lungs, mediastinum), another 30% in the peritoneal cavity, retroperitoneum, or pelvis, and approximately 20% occur in the head and neck, mainly in the sinonasal tract and oral cavity, while the rest are found in deep soft tissues of the trunk, extremities, or rarely in bone [4]. Because they can manifest anywhere in the body, the clinical presentation varies from painless mass to pulmonary symptoms or pain, according to its location [4-7]. The diagnosis is based on imaging studies and histologic examination. The treatment for patients with metastatic and locally advanced unresectable tumors includes chemotherapy and/or radiotherapy [2-4].

This case highlights a young patient who consulted her family physician (FP) due to a recently noted painless mass in the dorsal region, associated with low back pain radiating to the lower limb and refractory to analgesic therapy, who was later diagnosed with an aggressive metastatic SFT.

## Case presentation

A 30-year-old female patient, previously healthy, presented to her health center (HC) on September 27, 2023, after noticing a recent painless nodular lesion in the dorsolumbar region. Moreover, she complained about left-sided low back pain radiating to the ipsilateral foot with associated paresthesia with one week of evolution and no recent trauma. She denied systemic symptoms (including fever, weight loss, fatigue), the use of corticosteroids or other immunosuppressants, muscle weakness, and urinary or fecal incontinence. The pain improved partially with non-steroidal anti-inflammatory drugs (NSAIDs) and muscle relaxants. On physical examination, a hard lump was identified on the left dorsolumbar region, and she had a negative Lasègue sign. She reported a previous history of self-limiting pain three months ago in the same region where the lump appeared following physical activity. At that time she went to the emergency room, but the physical examination and the X-ray didn't show any anomalies, prompting a diagnosis of muscle pain due to overexertion. Taking that into account, the lump was now suspected to be a Grynfeltt-Lesshaft hernia, and the lower back pain was non-complicated, so a soft tissue ultrasound was requested, and analgesic and muscle relaxant medications were adjusted.

She returned to her HC on October 11, 2023, due to worsening pain, refractory to the prescribed therapy, now constant, and with bilateral hip pain impacting sleep and worsening when lying down. Given the worsening of the symptoms with associated low back pain red flags, a lumbar CT scan and blood work were requested, and the analgesic medication was adjusted, replacing the NSAIDs with an opioid analgesic every eight hours and pregabalin 75 mg every 12 hours. 

**Figure 1:**
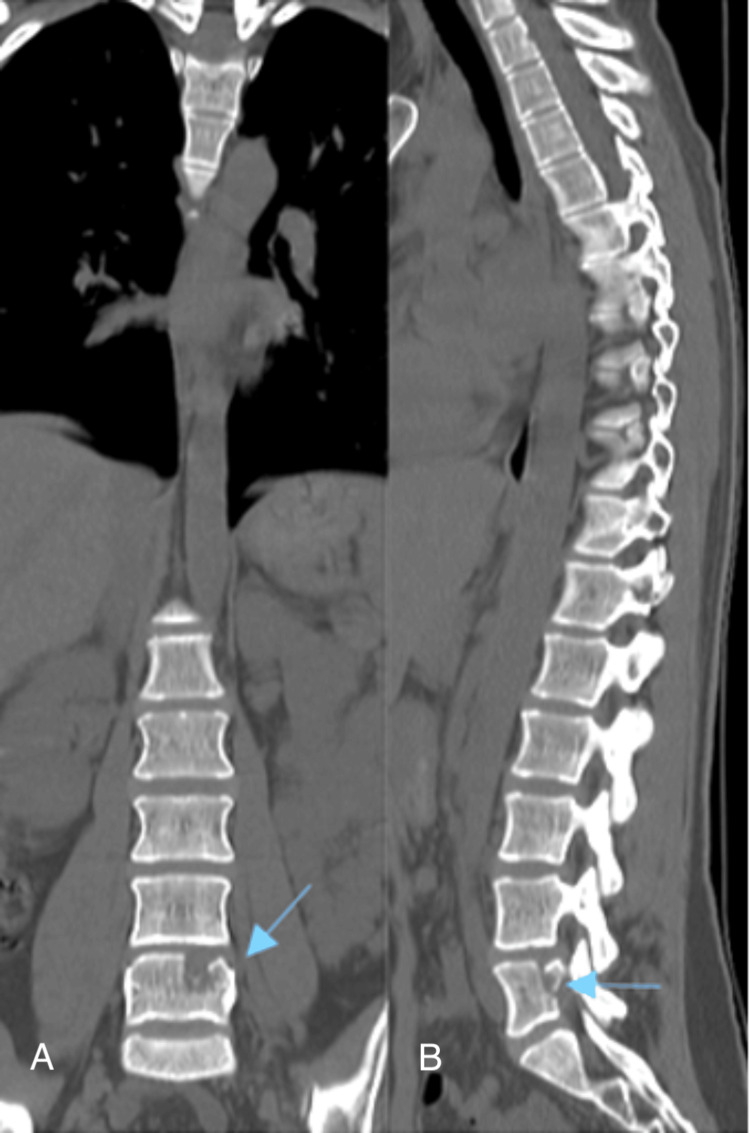
Lumbar CT scan A: coronal; B: sagittal views. The blue arrows indicate bone depression and deformation at the L4/L5 level.

**Figure 2:**
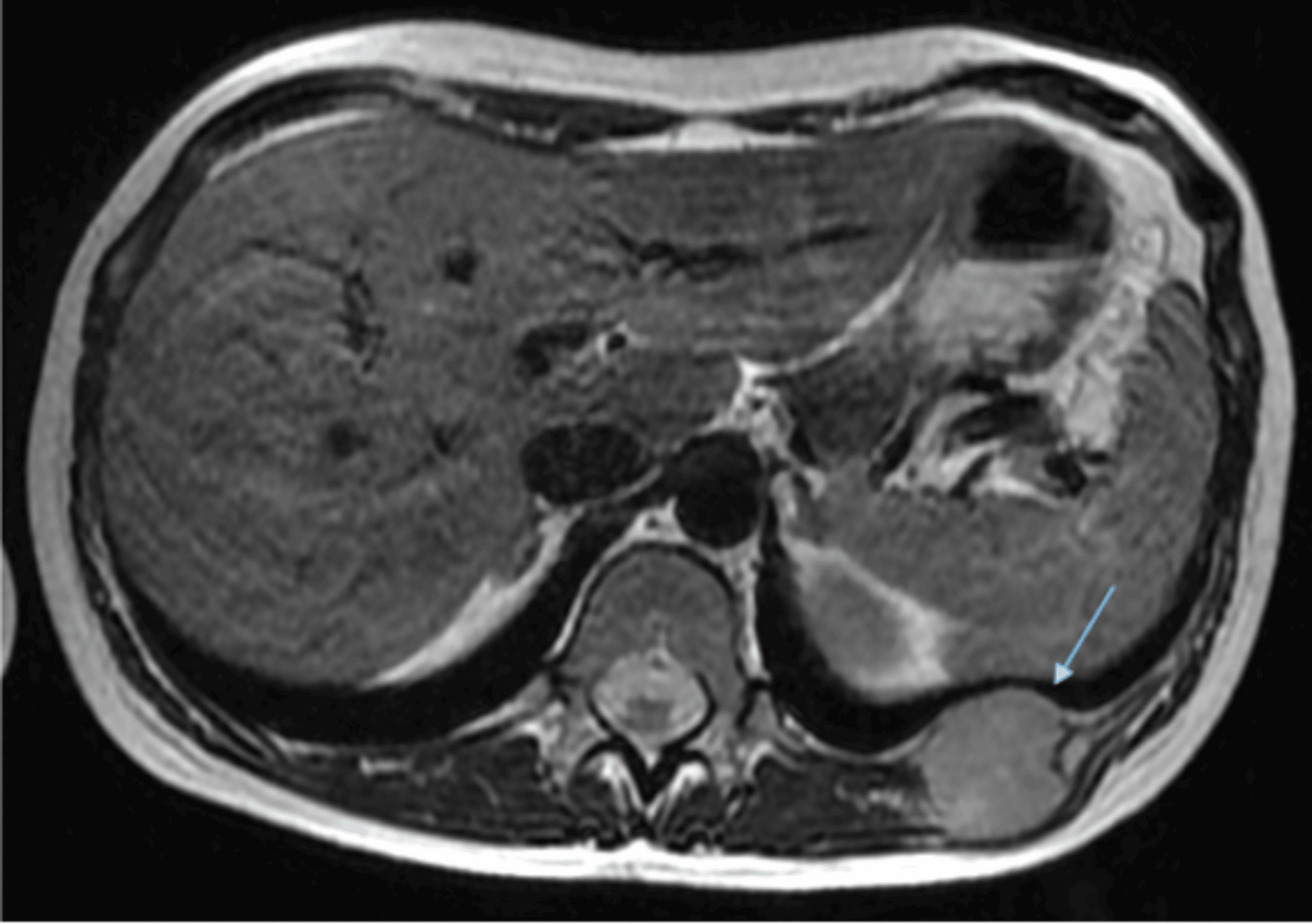
Thoracic MRI The blue arrow indicates a well-defined oval lesion located on the 11th left costal arch showing slight hypersignal (which points to the aggressiveness of the injury).

**Figure 3:**
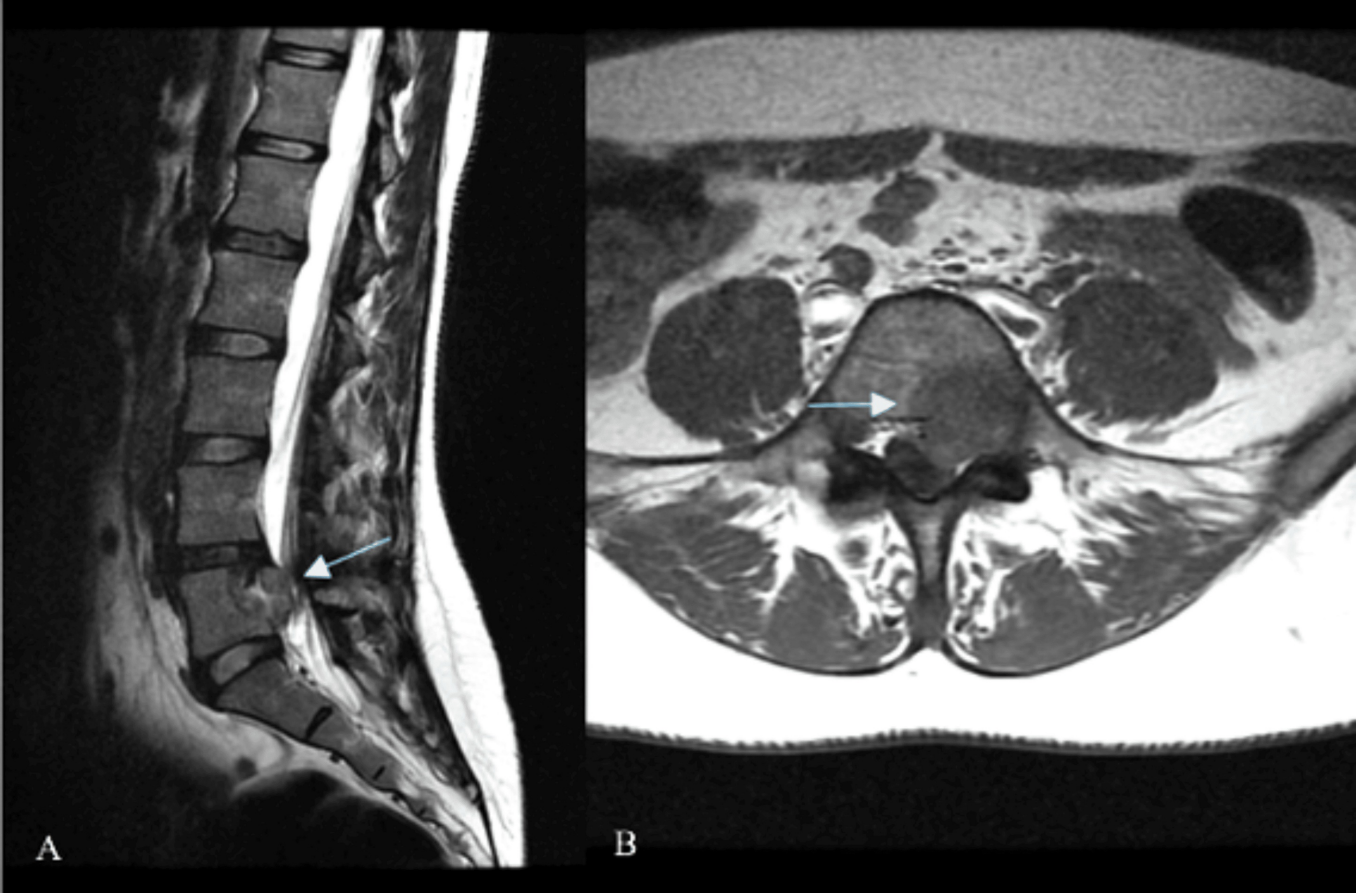
Lumbosacral MRI A: sagittal; B: axial views. The blue arrows indicate a lesion in the L5 vertebral body extending into the anterior and left margin of the central spinal.

One week later, she visited her FP to show the test results. The soft tissue ultrasound of the left dorsolumbar region reported a cystic lesion with calcifications recommending a radiological study of the chest and rib cage, and the lumbar CT scan (Figure 1) revealed bone depression and deformation at the L4/L5 level, raising concerns about a possible left posterolateral L4/L5 disc herniation with marked downward migration or spondylodiscitis with a compressive component recommending an MRI of the lumbosacral spine. Blood tests showed increased erythrocyte sedimentation rate and creatine phosphokinase with no other analytical changes. With the aim of expediting the process, and given that, in Portugal, an FP cannot request an MRI exam, the results were discussed with the patient, who decided to undergo private lumbosacral and thoracic MRI the next week. The thoracic (Figure 2) and lumbosacral MRIs (Figure 3) revealed a well-defined, aggressive-looking lesion at the 11th left rib and a neoplastic lesion on the L5 vertebral body extending into the spinal canal, suggesting the most likely diagnostic hypothesis as multiple myeloma, prompting the request of urgent blood tests and urgent referral to hematology by her FP.

**Figure 4:**
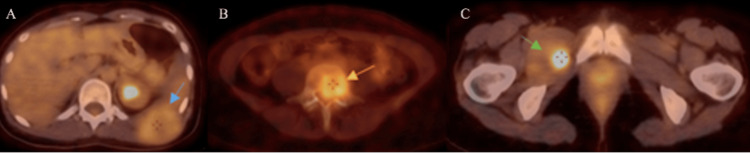
Body PET scan Hypermetabolic lesions on the 11th left costal arch (A: blue arrow), on the L5 vertebral body (B: orange arrow), and on the right femur (C: green arrow) suggestive of malignancy.

Upon evaluation by hematology, two weeks later, multiple myeloma was considered unlikely due to the absence of typical findings such as anemia, hypercalcemia, and monoclonal protein spikes. A biopsy of the dorsolumbar mass and a body PET scan (Figure 4) were requested, confirming the presence of a SFT and identifying hypermetabolic lesions in the thoracic region, L5 vertebra, and right femur, indicative of metastatic disease. She was discharged from hematology and referred to a sarcoma specialist.

Meanwhile, the FP contacted her for an update on the situation, and she reported uncontrolled pain with the previously prescribed medication. The analgesic medication was optimized with better pain control and doubts clarified.

On December 21, 2023, she had her first sarcoma appointment, where biopsies of the lesions in L5 and the right femur soft tissues were requested and performed on January 8, 2024, confirming the diagnosis of metastatic SFT.

On February 1, 2024, following a multidisciplinary meeting with oncology, she started palliative radiotherapy targeting the rib arch, right thigh, and L4/L5 lesions due to the L5 lesion being at risk of spinal cord compression, and one month later she started chemotherapy (pazopanib), with improvement in pain symptoms.

Currently, the patient is being treated with pazopanib and has close follow-up with oncology and sarcoma specialists, as well as her FP. Her pain is well-controlled, and she is awaiting a new PET scan to evaluate the effectiveness of the ongoing treatment.

## Discussion

SFT comprises a histologic spectrum of rarely metastasizing fibroblastic mesenchymal neoplasms. They are mostly benign and indolent, with low metastatic potential [2-4,8]. They can appear at any age, being more common between the fifth and seventh decades of life, with no gender preference [4,8]. SFTs are rare, corresponding to only 3.7% of visceral and soft tissue sarcomas, with an incidence of 0.35 per 100,000 individuals [4]. They preferentially arise in serosal membranes, the dura of the meninges, and deep soft tissues but can affect any part of the body [2,4]. 

Although there is an association with specific genetic alterations, particularly the fusion between the NAB2 and STAT6 genes [5,8], its etiology is not fully understood [2,4]. The clinical presentation varies according to the site of the tumor, from a painless mass to pulmonary symptoms or neuropathic pain [4,6,7]. The diagnosis must be suspected based on clinical examination and imaging tests (such as CT or MRI), and the definitive diagnosis must always be confirmed histologically. First-line treatment for localized disease is complete surgical resection; however, for locally advanced or metastatic tumors, chemotherapy is the recommended treatment. Radiotherapy can be used as an adjuvant for local control or with palliative intent [2-4]. The prognosis is favorable, with five-year survival rates between 59% and 100% and 10-year survival rates between 40% and 89% [2]. However, recurrence, even in tumors classified as benign, is common. Among malignant SFTs, 10-40% metastasize within five years of diagnosis, and relapses may occur up to 20 years after initial diagnosis, which highlights the need for long-term follow-up [4].

## Conclusions

The unusual presentation of this case, which involves a young patient with a metastatic SFT with rapidly worsening symptoms, highlights the importance of recognizing the red flags of low back pain and remembering that not all common symptoms are consequences of common diseases. 

It underscores the importance of the FP role in early detection, timely referral, and ongoing management of complex cases, participating in all phases of the disease. The fact that in Portugal FP cannot prescribe MRIs could have delayed the diagnosis, given the poor MRI availability in public hospitals. Fortunately, the patient had health insurance, so the referral from the family doctor, who was in charge of the initial diagnostic workup, to the hospital was swift. Through comprehensive assessment and appropriate use of diagnostic resources, family physicians can significantly impact patient outcomes in such cases.
